# Effect of a Child-Owned Poultry Intervention Providing Eggs on Nutrition Status and Motor Skills of Young Children in Southern Ethiopia: A Cluster Randomized and Controlled Community Trial

**DOI:** 10.3390/ijerph192215305

**Published:** 2022-11-19

**Authors:** Anteneh Omer, Dejene Hailu, Susan J. Whiting

**Affiliations:** 1 School of Human Nutrition and Food Science, Hawassa University, Hawassa P.O. Box 5, Ethiopia; 2 School of Public Health, Hawassa University, Hawassa P.O. Box 5, Ethiopia; 3 College of Pharmacy and Nutrition, University of Saskatchewan, Saskatoon, SK S7N 5E5, Canada

**Keywords:** egg, growth, one egg a day, poultry, chicken, stunting, wasting, underweight, gross motor skill

## Abstract

Eggs are highly nutritious foods, yet intake by children in Ethiopia is low. We hypothesized that a nutrition-sensitive poultry intervention improves nutritional status of children 6–18 months using a 6-month cluster randomized controlled community trial. Intervention group (IG) children received a gift of two egg-laying hens in a ceremony where children’s ownership of the chickens was declared by community leaders. Parents promised to add more hens and feed the owner-child one-egg-a-day. Trained community workers reinforced egg feeding, environmental sanitation and poultry husbandry. Control group (CG) mothers received usual nutrition education on child feeding. At baseline 29.6% of children were stunted, 19.4% underweight and 8.6% wasted. Egg consumption significantly increased only in IG, at 6 months. The intervention increased weight-for-age and weight-for-height z-scores by 0.38 (95% CI = 0.13–0.63) and 0.43 (95% CI = 0.21–0.64), respectively. Binary logit model indicated IG children were 54% (Odds ratio [OR] = 0.46; 95% CI = 0.26–0.84) and 42% (OR = 0.58; 95% CI = 0.37–0.91) less likely to be underweight and stunted, respectively, compared to CG. IG children attained the milestone of running (*p* = 0.022; AHR = 1.43; 95% CI = 1.05–1.95), kicking a ball (*p* = 0.027; AHR = 1.39; 95% CI = 1.04–1.87) and throwing a ball (*p* = 0.045; AHR = 1.37; 95% CI = 1.01–1.86) earlier than CG. This nutrition-sensitive child-owned poultry approach should be implemented where animal-source food intake is low.

## 1. Introduction

Undernutrition among children of under five years of age is a global public health concern with the highest burden in South Asia and Sub-Saharan Africa [1]. Ethiopia, the second most populous country in Africa located at the horn, recorded rates of stunting (37%), underweight (21%) and wasting (7%) above the regional average for East Africa [2,3]. Though a declining trend was observed in the past two decades, Ethiopia is off track with progress in meeting the 2025 global nutrition targets of the World Health Assembly particularly in child stunting and wasting [1,4,5]. If the current trend continues, the country will not meet Sustainable Development Goals target 2.2. by 2030.

Most of the malnutrition in Ethiopia starts after the age of six months when the children are introduced to complementary foods. Poor feeding practice is one of the important factors causing undernutrition among children under two years of age. Diets are generally cereals and lack fruits, vegetables and animal source foods (ASFs) [6]. Egg intake among Ethiopian children under two years is very low (17%) due to economic and other reasons [7]. Eggs are relatively affordable ASFs and can be made easily available through homestead poultry. Eggs are rich in various high-quality macro and micro-nutrients including protein, choline, riboflavin, vitamin B6, vitamin B12, folate, zinc and DHA as well as antioxidants and compounds beneficial for child health, nutrition, physical growth and brain development [8]. 

Evidence is limited on the effects of egg intake on health, nutrition, growth and development of infants and young children in low-income countries. Improved nutrient intake including protein, choline, B vitamins, lutein, zeaxanthin as well as many important minerals and overall energy intake was reported as a result of egg consumption [9,10,11,12,13]. Egg interventions have reported increased linear growth, underweight and wasting, although effects were not consistent in different settings. For instance, the results of an egg trial in Ecuador [14] could not be replicated in Malawi [15]. A reduction in underweight and anemia reported by a nutrition-poultry interventional study in Ethiopia [16] was not found in a similar community trial in Burkina Faso; rather a reduced wasting rate was recorded [17]. Only one study was found that reported the effect of egg consumption on developmental outcomes. Intake of one egg a day did not affect language, fine and gross motor milestones of young children in Malawi [18].

Backyard poultry can play an important role in increasing egg intake and thereby help improve health and nutrition of infants and young children particularly in rural settings [19,20,21]. However, poultry husbandry in most rural communities of less developed countries consists of chickens living in the family house and scavenge in the surrounding environment for food [22]. Studies have associated corralling of chickens with linear growth faltering in children as a result of enteric infection called environmental enteric dysfunction (EED) that arises from repeated exposure to bacteria including Campylobacter species from the birds and their feces [23,24]. In EED, nutrients are poorly absorbed due to atrophied villi and lost due to increased intestinal permeability (leaky gut) [25]. Thus, poultry interventions that aim increased egg intake and improved health and nutrition status should consider strategies to reduce disease transmission from chickens to children [26].

Our pilot trial of nutrition-poultry integrated intervention that declared chicken ownership by children and promoted use of separate chicken areas recorded a significant increase in egg intake [27] and a reduced underweight rate [16] by young children. We now report on a nutrition-sensitive poultry intervention following that approach of having a chicken gift ceremony model but includes provision of caging materials to minimize direct contact of children with the birds and their excreta. The first objective of our study was to evaluate the effect of child-owned poultry-nutrition intervention on egg intake and poultry husbandry practices, which we have recently published [28]. Therein we reported that mean egg intake significantly increased in the treatment arm (from 0.23 to 4.85 eggs/child/week) while only slightly changed in the control arm (from 0.29 to 0.40 eggs/child/week) at end line [28]. We also had the objective to assess whether promotion of egg consumption and proper poultry husbandry through child-owned poultry-nutrition intervention would positively affect growth and developmental outcomes. This paper presents anthropometry and gross motor milestone outcomes of our child-owned poultry-nutrition intervention to address this second objective.

## 2. Materials and Methods

### 2.1. Study Design, Participants and Sample Size

The study is a cluster-randomized community trial conducted in rural villages of Halaba district, Southern Ethiopia, from May to November 2018 (ClinicalTrials.gov: NCT03355222). Halaba has ten catchments, each one having a health center that provides technical support for health posts in each village (kebele). At the community level, health and nutrition activities are mainly led by health extension workers (HEWs) who are supported by health development team leaders (HDTLs) who are connected to 30 households. The district is recurrently drought affected and endemic to malaria.

Two out of ten existing clusters in the district were randomly selected and allocated to contain the intervention group (IG) and the control group (CG). Then, a village was selected from each selected clusters purposely for matching reasons paying particular attention to the distance from catchment health center and existing nutrition and agriculture interventions. It was also ensured that the two villages did not share borders or have a common local market to minimize information contamination. Considering effect size of 0.5, power of 80%, design effect of 2 and 10% loss to follow up, a sample size of 126 mother–child pairs was calculated for each study group. Children of 6–18 months old who had lived in the area at least for six months were eligible for enrollment. Those who were reported by their caregivers to be allergic or sensitive to egg, sick and taking drugs, on nutritional treatment, or severely and acutely malnourished were excluded from the study. As a result, all the remaining children from the intervention kebele (n = 127) and from the control kebele (n = 126) were enrolled in the study [28]. 

Different groups of data enumerators were deployed at baseline and end line. Participants, health extension workers (HEWs), agriculture extension workers (AEWs) and health development team leaders (HDTLs) in both study villages were not told about which group they were in and even the existence of two groups. The anthropometrist was also blind to study groups. Details of sampling procedure, sample size determination, participants and study protocol are published [28].

### 2.2. Intervention

A series of intervention activities were implemented in IG utilizing the existing government personnel structure from district to community level. The intervention started by building the capacity of front-line implementers working in the kebele, including HEWs, HDTLs and AEWs. They received training on importance of egg consumption of children, benefits of separate chicken shelters (caging) and proper poultry husbandry emphasizing cage utilization, and danger of chicken feces. They were apprised of their respective roles in the research project. HEWs, AEWs and HDTLs in the control had received only the usual nutrition and agriculture training. However, training similar to the intervention kebele was provided to them after the end-line surveys. 

#### 2.2.1. Chicken and Caging Gift Ceremony Model

The study designed and implemented a unique approach called the Chicken and Caging Gift Ceremony model (CCGC) for sustainable and increased egg intake. CCGC aims toward the consumption of one egg a day in infants and young children by enabling them to be owners of egg-laying chickens through culture- and religion-sensitive community-based ceremonial approach, as described previously [28]. In brief, after providing training to HEWs, HDTLs and AEWs and orientation to community and religious leaders in the intervention kebele, a cultural and religious ceremony was organized where the children received two egg-laying local breed hens with caging materials. In the event, religious and community leaders declared that the children were owners of the chickens and all the eggs the birds produce, and selling or sharing of the eggs, as well as the chickens, is ‘Haram’ (Arabic word meaning ‘forbidden’). Caregivers promised to take care of the chickens, present at least two hens as a gift, replace them when the birds die, not to sell nor share the eggs and to feed the chicken owner child one egg every day.

Two types of cages provided were previously described [28]. The night coop was 70 cm high, 100 cm long and 80 cm wide with 5 cm high legs. It was framed in wood, covered with mesh wire on its four sides for ventilation and finished with waterproof floor material that can come out and fit back for easy cleaning. It had two perches inside, 0.80 m long each, that up to eight chickens could roost on. For the day enclosure, parents received eight wood logs of 1.5 m long and 0.10 m wide, 20 m of mesh wire, and nails. As we reported, over 90% of IG families used the night coop and 86% kept chickens in a separate area using the day enclosure caging. 

#### 2.2.2. Nutrition and Poultry Promotion

After CCGC, the HEWs conducted demonstration of preferred egg cooking methods which were selected taking food safety, swallowing ability of the children and simplicity of preparation into consideration. These were hard boiled and smashed egg yolk for age of 6–7 months and hard boiled and smashed whole egg for children aged older than 7 months. Mothers were told to express breast milk to soften the smashed egg for easy swallowing. In addition, they were informed to encourage the child to finish at one sitting in order to avoid feeding of leftover egg later as it might be contaminated. 

The HEWs and HDTLs passed along social and behavioral communication change messages of promoting one egg a day consumption, child-owned poultry production and proper poultry husbandry emphasizing on environmental sanitation using counselling cards prepared for this purpose. HEWs provided individual counselling based on weight for age results promoting one egg a day consumption and baby friendly child-owned poultry practice. HEWs and HDTLs used any possible contact with caregivers to conduct nutrition and poultry promotion including home visits and when caregivers came to the health post for any reason, for example, data collection. 

Trained AEWs promoted one egg a day consumption and proper poultry husbandry practice encouraging child-owned poultry production and emphasizing cage utilization. They showed the caregivers how to utilize and clean the night coop as well as prepared a prototype of day enclosure caging. AEWs counselled caregivers individually every month using cards specially prepared for this purpose based on findings of poultry production and practice data. In addition, they promoted environmentally safe baby friendly poultry practice when they visited households for assessment of poultry husbandry practices.

### 2.3. Data Collection and Measurements

Intervention effect on nutritional status of the children was primary outcome while time of attainment of selected gross motor milestones was measured as a secondary outcome. Other outcomes, not reported here, include effects on anemia, hemoglobin and morbidity. Pre-intervention anthropometry, motor skills, complementary feeding practice and socioeconomic and demographic data were collected in May 2018.

For anthropometry assessment, weight and mid upper arm circumference (MUAC) measurements were taken every month while length was taken at baseline, mid line and end line. A trained and experienced nutritionist took all anthropometry measurements based on the standard procedures outlined in the anthropometric indicator measurement guide of WHO/UNICEF [29]. Weight (tared), MUAC and recumbent length (recumbent) were measured twice, and average was taken. When a difference of more than 0.2 units was found between two measurements, a third measurement was taken. Weight measurement was carried out using electronic scale (Seca 874, Hamburg, Germany) while the children were in their caregivers’ lap and after weight of the caregivers and children’ cloths was made zero. Length was measured with Seca 417, Hamburg, Germany without hat and shoe. 

Gross motor milestones were taken after two days of practical training with testing, given to 60 health development team leaders (HDTL). Of these, there were 32 in the IG and 28 in CG who carried out assessment of gross motor milestones every two weeks. On average, one HDTL was assigned to five children for this task. The motor milestones included in the assessment were sitting without support, standing with assistance, hands and knees crawling, walking with assistance, standing alone, walking alone, running, kicking a ball forward, throwing a ball overhead and jumping up. The steps described in WHO [30,31] and the revised Denver Developmental Screening Tool (Denver II) [32] were closely followed during training and assessment. A milestone was recorded as achieved only when the HDTL observed the child successfully performing the activity of interest. Caregivers’ verbal report of performing a milestone was not taken into consideration to avoid bias. Standardized criteria were utilized for assessing each developmental skill. Examinations were carried out at children’s homes, independent of all previous assessments.

Malaria testing (Plasmodium vivax and Plasmodium falciparum) was provided for all children at baseline and end line. Two medical laboratory technologists performed the test from finger prick blood samples using the rapid diagnostic test CareStart™ Malaria HRP2/pLDH (Pf/Pv) Combo, Kit/25 according to the manufacturer’s instructions (Access Bio Inc., Addis Ababa, Ethiopia) at health posts of the study kebeles. 

Intestinal helminthiasis examination was carried out from randomly selected sub-samples of 50 children from each group. A gram of stool was emulsified in 4 mL of 10% formol water in a plastic stool cup, screwed tightly and transported to Halaba General Hospital Laboratory Parasitology Unit. The samples were analyzed using Formol-ether concentration technique based on the standard operating procedures outlined by Cheesbrough [33]. The emulsified fecal suspension was strained using gauze; 10 mL of 10% formol water was added and centrifuged for 1 min. After decanting the supernatant, 8 mL of formol water and 4 mL of diethyl ether were added and centrifuged. The supernatant was decanted, and the sediment was examined microscopically for intestinal helminths with egg count.

### 2.4. Statistical Method

Data entry and analysis were made using IBM SPSS version 28 (Chicago, IL, USA). Baseline characteristics were presented by descriptive analysis with a comparison between the study groups by cross-tabulation/Pearson chi-square and independent samples *t*-test for categorical and continuous variables, respectively. Paired *t*-test was used to compare baseline and end line anthropometric z-scores. Trend analysis and generalized estimating equations (GEE) were applied to assess the intervention effect on the nutritional status of the children. GEE was preferred method considering the design of the study (cluster-randomized), repeated measurements taken, and the presence of some missing values observed at different times during the intervention period. GEE linear and binary logistic regression models with autoregressive correlation matrix and robust estimator were run and effect sizes were presented as beta and odds ratio.

The intervention effect on the achievement of gross motor development milestones was analyzed using the Kaplan–Meier survival analysis model with log-rank for comparison of age of attainment between study groups. Effect sizes were computed as crude and adjusted hazard ratios by Cox regression. WHO Anthro Survey Analyzer was used to develop figures of anthropometric z-scores distribution curves and excel was used to prepare graphs [34]. A *p*-value less than 0.05 was considered statistically significant in all analyses.

### 2.5. Ethics

The study was approved by Hawassa University Institutional Review Board, Ethiopia and the University of Saskatchewan, Canada. Necessary communication was made with Halaba district administration, and health and agriculture offices through a letter written by the School of Nutrition, Food Science and Technology, Hawassa University. Heads of catchment health centers, kebele managers, kebele chairpersons and community and religious leaders of both study kebeles were approached before commencing research activities. Caregivers gave their consent by signing to participate in the study after explaining the purpose. They were also informed about the lab tests and procedures before taking blood and stool samples. Participants were given a bar of soap and oil as compensation for their time during the follow-up. Children who were excluded from the study due to caregivers’ reports of egg sensitivity were appreciated by giving them chickens. At end line, children in the control group received two egg-laying hens along with passing chicken gift ceremony messages to families.

## 3. Results

The average compliance rate for data collection was above 93% in a given month during the study period. Excluding those who were lost to follow-up (7) and found to be sensitive to eggs (3), a total of 243 children entered into the analysis of intervention effect on nutrition status and gross motor milestones.

### 3.1. Baseline Characteristics

The study groups were not different in most indicators including infant and young child feeding (IYCF) practices (Table 1). Baseline characteristics are presented in more detail in [28]. 

### 3.2. Intestinal Helminthiasis and Malaria Infection

No child was infected with intestinal helminths at baseline. At end line, 7% of the children were infected with intestinal helminths with no statistical difference between the study groups (*p* = 0.436; Pearson Chi-square = 1.382). No child was found with multiple helminth infections. The most prevalent helminth was *Hymenolepis nana* (egg count ranging from 2 to 19 eggs/gram of feces) followed by Ascaris lumbricoides that infected only one child in the control group (220 eggs/gram of feces). All children were negative for malaria (*P. falciparum* and/or *P. vivax* infection) at baseline and end line in both groups.

### 3.3. Anthropometric Indicators

Both groups were comparable in their pre-intervention nutritional status (Table 2). About 29.6% of the children were stunted (8.6% severe stunting); 19.4% were underweight (3.7% severe underweight) and 8.6% were wasted. Except for weight-for-height z-scores (WHZ), mean scores of weight-for-age (WAZ) and length-for-age (LAZ) (*p* < 0.001) decreased at end line in the control group compared to their corresponding baseline values. In contrast, mean WAZ, LAZ and WHZ scores increased significantly in the intervention group. The difference in anthropometric indicators among the study groups was also observed in the distribution curves of z-scores, particularly WAZ and WHZ that shifted remarkably to the right at end line among children in the intervention arm (Supplemental Figure S1). Underweight and wasting decreased significantly at end line in the intervention arm whereas only wasting showed a reduction in the control. Stunting increased in both groups but was significant only in the control. 

**Figure 1:**
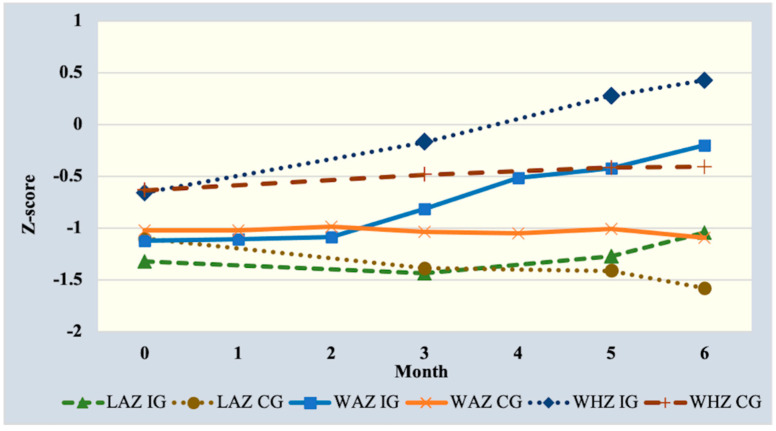
Trend of length-for-age (LAZ), weight-for-age (WAZ) and weight-for-height (WHZ) Z-scores from baseline to end line over 6 months. IG = intervention group; CG = control group.

Generalized estimating equation linear modeling showed that weight for age and weight for height z-scores increased significantly by 0.38 (CI: 0.13–0.63) and 0.43 (CI: 0.21–0.64), respectively, in the intervention group at end line, but not LAZ, compared to the control (Table 2). However, trend analysis showed that LAZ in the intervention group increased after the third month of the intervention period until the end while deteriorating in the control arm throughout the study period (Figure 1). Results were comparable when different correlation matrices were entered into the model except for effect size on WAZ (0.27 (CI: 0.02, 0.52) (Supplemental Table S1).

**Figure 2:**
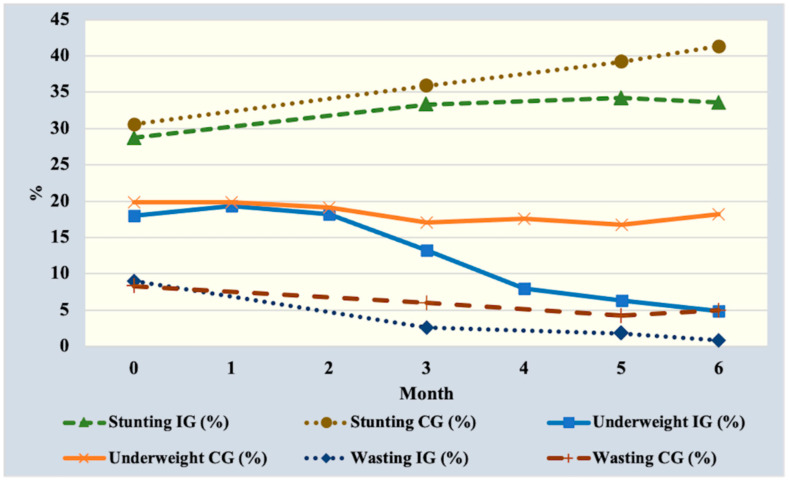
Trend in proportion of children in each nutritional status group from baseline to end line, over 6 months. IG = Intervention group; CG = control group.

Children in the intervention group were 54% and 42% less likely to be underweight and stunted at end line compared to the control (Table 2). Child age, sex, maternal and household factors were entered into the GEE models (as covariates and cofactors) and not found to have a significant effect. Trend analysis revealed that the intervention affected underweight sooner than stunting (Figure 2). Underweight started to decrease starting from the third month of intervention. Stunting continued to increase, yet the pace slowed after the third month of the intervention period, after which, there was a declining trend to end line.

### 3.4. Gross Motor Milestones

About 7.1% and 17.4% of the children were at the stage of standing with assistance (*p* = 0.151) and crawling (*p* = 1.00), respectively, during the baseline survey. Nearly one out of four children were able to walk with assistance (*p* = 0.883) and stand without support (*p* = 0.658). One-fifth of them were capable of walking without assistance (*p* = 0.437). In addition, 7.5% of the children demonstrated the motor skill of running correctly (*p* = 0.244). No child was able to kick a ball, throw a ball or jump during the baseline survey. At the endpoint, children in the intervention attained milestones of running (*p* = 0.04) and kicking a ball (*p* = 0.042) earlier (17.50 ± 1.71 and 18.90 ± 1.71 months of age, respectively) than those in the control (17.86 ± 2.18 and 19.26 ± 2.30 months, respectively). Compared to the control, an increased probability of achieving skills of running (AHR:1.32; 95% CI: 1.01–1.73; *p* = 0.044) and kicking a ball (AHR:1.30; 95% CI: 1.001–1.692; *p* = 0.049) was observed in the intervention group. 

The intervention effect on gross motor milestones was more pronounced among normal-weight children (WAZ ≥ −2.0) at baseline. After adjusting for baseline stunting or comorbidity of anemia and stunting, children in the intervention arm achieved motor skills of running (*p* = 0.021), kicking a ball (*p* = 0.027) and throwing a ball (*p* = 0.046) at lower ages (17.53 ± 1.70, 18.96 ± 1.73 and 20.90 ± 1.31 months, respectively) than those in the control (17.96 ± 2.26, 19.41 ± 2.38 and 21.18 ± 1.64 months, respectively). They also had 1.43 (*p* = 0.022; 95% CI: 1.05–1.95), 1.39 (*p* = 0.027; 95% CI: 1.04–1.87) and 1.37 (*p* = 0.045; 95% CI: 1.01–1.86) times increased probability of attaining the aforementioned milestones in their respective order compared to children in the control (Table 3).

**Figure 3:**
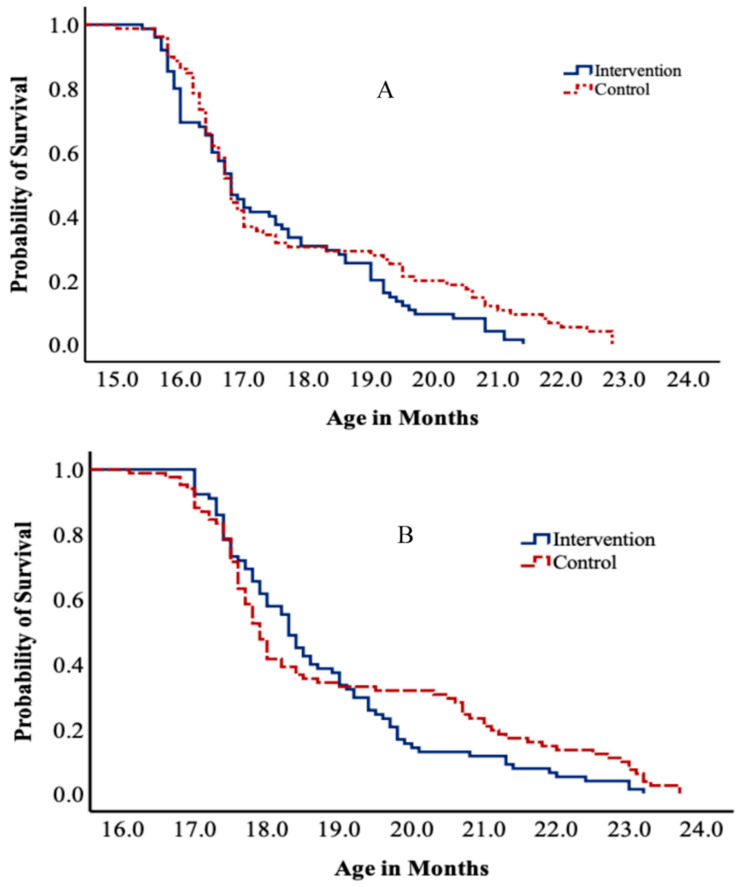
Kaplan–Meier curves of running (**A**) and kicking a ball (**B**) motor skills attainment among normal weight children at baseline in intervention and control.

Survival curves revealed the time of attaining running and kicking ball milestones showed a prominent difference between intervention and control groups among children who achieved the skills at a later age (after 18 and 19 months) (Figure 3). 

## 4. Discussion

A nutrition-sensitive poultry intervention in which 6–18 months old children were owners of chickens was conducted through an innovative community-based approach. The intervention resulted in increased chicken production and improved poultry husbandry practices including cage utilization and vaccination. It also progressively and sustainably increased egg intake from 0.23 to 4.85 eggs/week/child, dietary diversity score from 2.34 to 3.34 and the proportion of children who met the minimum dietary diversity from 4.7% to 37% at end line [28]. This paper focused on the effects of the intervention on the nutritional status and gross motor development of the children. 

Baseline stunting, underweight and wasting rates were comparable with that of the Ethiopian mini demographic and health survey report among 6–23 months children [2]. After six months of intervention, adjusted GEE analysis showed the children’s WAZ and WHZ increased by 0.38 (95% CI: 0.13–0.63) and 0.43 (95% CI: 0.21–0.64), respectively compared to the control. In addition, the odds of underweight and stunting decreased by 54% (OR: 0.46; 95% CI: 0.26–0.84) and 42% (OR: 0.58; 95% CI: 0.37–0.91), respectively compared to control. These findings are much higher than what we reported in our pilot study [16]. A similar effect on WAZ and WHZ was recorded by a 3-arm controlled trial of nutrition-poultry intervention in Burkina Faso that stressed children’s ownership of chickens. The full intervention arm received nutrition-poultry education and chickens while the partial one received only the behavior change package. Egg intake increased from 0.1 to 6.3 eggs per week accompanied by an increase in WAZ and WHZ by 0.47 and 0.58, respectively among children in the full intervention group. The partial arm had an increase in egg intake from 0 to 2.4 eggs per week that resulted in a lesser positive effect on nutritional status which was not significant [17]. 

The increase in WAZ and WHZ recorded in this trial was four and five times, respectively bigger than what was found through supplementation of eggs to 6–12 months old children in rural Bangladesh [35]. One-egg-a-day supplementation in Ecuador increased WAZ, WHZ, and LAZ by 0.61, 0.33 and 0.63, respectively and reduced stunting and underweight by 47% and 74%, respectively; the biggest effect sizes reported so far from egg interventions [14]. However, these results were not replicated in the Malawi egg trial, a successor of the Ecuador study with a similar design, probably due to high fish intake in the study area [15].

Despite the increase in mean LAZ score compared to the baseline and control group, stunting increased at the end line. However, our intervention demonstrated a protective effect against stunting. The increasing pace of stunting was substantially slowed and the odds of being stunted were significantly reduced. The protective effect of egg intake against stunting was also reported in our pilot study [16] and the Burkina Faso trial as stunting increased less in the full intervention arm than in the partial and control groups [17]. The intervention effect on stunting was also supported by a nutrition-sensitive agriculture study conducted recently in Ethiopia that attributed the increased LAZ score and reduced stunting to sustainable access to egg consumption [36]. 

The longitudinal analysis did not find significance in LAZ. However, this did not mean the intervention did not affect linear growth. Trend analysis revealed that LAZ respond to our intervention in the last two months of the study. It increased first in the fifth month and continued to the end line with a significantly higher score than the control. Several factors might have attributed to this result including the dose of egg consumption and study duration. Children in the intervention had relatively higher egg consumption during the last three months of the study period [28]; yet did not reach the level of one egg a day which was the dose used in the Ecuador trial that recorded greater results [14]. The six months study duration combined with such a trend and dose of egg intake might not be sufficient to see the effect on linear growth as was seen in a study in rural Zambia [37]. Had the intervention been implemented for longer, a greater effect of child-owned nutrition-sensitive poultry on linear growth and stunting reduction might have been demonstrated.

Apart from anthropometric indicators, this study recorded a remarkable effect on the gross motor development of intervention children. Notably, running, kicking, and throwing ball milestones were attained significantly at an early age compared to control. Low nutritional status and anemia are associated with delayed motor development [38,39,40]. Both groups were delayed in attaining hands and feet crawling, walking with assistance, standing alone and walking alone milestones compared to the WHO Multicenter Growth Reference Study [30] and a longitudinal study in India on the development of children in Kashmir in the first 2 years of life [41]. The intervention effect was magnificent in attaining motor milestones, particularly running, kicking a ball and throwing a ball compared to the control. The children attained these skills at lower mean ages (17.53 ± 1.70, 18.96 ± 1.73 and 20.90 ± 1.31) in their respective order) compared to the India study (17.87 ± 2.03, 19.63 ± 3.26, 20.94 ± 3.35, respectively). Though not significant compared to the control, intervention children achieved the jump-up milestone (23.42 ± 0.7) at an earlier age than the control (23.5 ± 0.83) and Kashmir children in the Indian study (23.60 ± 1.25). Children in the control were late in attaining the running and throwing a ball and a little early in the kicking a ball and jumping up milestones compared to Kashmir children [41]. The rates of attaining motor milestones found in this study were much greater than what was recorded in the trial of water, sanitation, handwashing (WASH) and nutrition intervention in Bangladesh and Kenya [42,43].

Taking the age of the children at enrollment into consideration (mean age: 10.9 and 11.4 months in intervention and control groups, respectively), the first four milestones were achieved soon after enrollment in the study by the time the children’s egg intake was low, at least not sufficient enough to have an effect on motor development, though improved from the baseline. Our intervention showed a remarkable effect on those milestones that the children achieved after they had higher egg intake for longer periods relatively supporting the evidence base that diet has a significant contribution to children’s motor development. Nutrition factors including complementary feeding frequencies, dietary diversity, and egg and oil intake were significant predictors of earlier achievement of motor and language development outcomes among young children in Haiti [42]. Rate of attaining motor and other developmental milestones increased when the nutrition component (lipid-based nutrient supplementation and IYCF counselling) was added to the water, sanitation and handwashing (WASH) intervention in Bangladesh [43]. Meat and other types of ASFs consumption in Nepal [40], dietary diversity and intake of iron-rich foods in Bangladesh [44], intake of lipid-based nutrient supplements in Ghana (LNSs) [45] and Burkina Faso [46] and home fortification with LPS and/or micronutrient powder in Bangladesh [47] were associated with better motor and other developmental milestones. On the contrary, the LNS trial in Malawi, a replica of the Burkina and Ghana studies, showed only a marginal effect [48]. Provision of one egg a day also had no effect on child development in Malawi [18]. This happened probably due to chance as explained by both studies, high fish intake as stated by the egg trial or other unknown factors.

The improvement in the nutritional status of the children in this study might have contributed to their motor development as there is a positive relationship between nutritional status and gross motor skills. The significant contribution of nutritional status on gross and fine motor skills development was revealed by a study in Indonesia that nutritional status explained 41.8% of child’s gross and fine motor skills with a correlation value of 0.650 [49]. Increased weight-for-age [38,50], height/length-for-age [38,40,50,51] and weight-for-height/length [38,40] were associated with and found to be important predictors of motor and other domains of development including language skills.

Eggs provide several nutrients critically essential for early growth and development. Studies reported that egg intake was associated with higher intakes of total protein, total fat, monounsaturated fat, polyunsaturated fat, cholesterol, phosphorous, Vit B12, D and E, pantothenic acid, biotin, choline, lutein and zeaxanthin, α-linolenic acid and docosahexaenoic acid [13,52]. Prevalence of inadequacy was also found to be lower among egg-eaters than non-egg-eaters for Vit A, E, D, B5, B12, riboflavin, zinc, iron, selenium and choline [52,53]. However, there are several nutrients such as vitamin c, B vitamins, calcium and iron that are not found in high quantities in eggs; thus, need to be fulfilled by other sources [52,53].

Although all nutrients are important for growth and development, nutrients particularly protein; zinc; iron; choline; folate; iodine; vitamins A, D, and B_12_; and long-chain polyunsaturated fatty acids including docosahexaenoic acid and arachidonic acid play an indispensable role in neurocognitive development [54,55,56]. Early infancy deficiency of these nutrients is associated with long-term neurobehavioral damage that may not be reversible by nutritional therapy [57]. On the contrary, early supplementation is associated with improved neurodevelopmental outcomes over an extended period of life. Children in Guatemala supplemented with a high protein high-calorie drink at an early age recorded higher scores on tests of general knowledge, numeracy, reading, and vocabulary along with faster reaction time in information-processing tasks than those provided with no protein low-calorie drink eleven years after the intervention stopped [58]. A choline supplementation study among children with fetal alcohol spectrum disorder also found significant neurodevelopmental benefits including non-verbal intelligence, higher visual-spatial skills, higher working memory ability and better verbal memory compared to the placebo group after four years of the trial completion [59]. Eggs are excellent sources of protein, choline, Vit B12 and long-chain polyunsaturated fatty acids. Their contribution of zinc, folate and Vit D to the daily requirements of infants is also remarkable. Thus, the consumption of one egg a day at an early age of complementary feeding probably might have long-term benefits in neurodevelopment besides the reported positive short-term outcomes on growth and motor development.

We deemed the improved poultry husbandry practices documented in the intervention group, particularly the cage utilization [28] magnified the effects recorded on child growth and development. In our pilot study in which we gave education on the danger of chicken feces promoting separate chicken shelters and sanitation of the household environment, we found encouraging results on child health and nutrition outcomes through increased egg intake, but effect sizes were less than what we found in this trial [16,27]. In the current study, after the provision of caging materials, keeping chickens in a shelter separated from the family house or inside a night coop placed in a corner of the family house was widely practiced. The night cage was movable, easily cleaned and small in size so that children could not climb inside, unlike the one used in the Peru study which was installed in the family house causing accumulation of chicken feces and the probable entrance of children into the coral [60]. The chickens were kept in an enclosure during the day that, together with the night coop/separate chicken shelter, might have minimized children’s contact with the chickens and their excreta. However, we did not collect information on exposure of children to chicken feces. Chickens are important reservoirs of Campylobacter bacteria that cause enteric infection in children. Studies reported an association between poultry keeping with stunting highlighting the importance of keeping chickens in separate shelters or cages [23,26]. 

Taking the benefits of egg intake in improving early childhood growth and development into consideration, homestead poultry is a recommended intervention particularly in rural settings to increase egg availability for consumption. When integrated with nutrition education, backyard poultry, regardless of the flock size, plays a great role in increasing egg intake sustainably among children [61]. The Ecuador trial result was no more found after two years as only a few households continued the practice of egg feeding because the intervention was based on egg supplementation [62]. Our intervention gave due emphasis to the sustainability of child-owned chicken production and egg-feeding practice which is the strength of this study. Existing government structure and personnel from the district to community level were utilized; religious and community leaders were engaged from the beginning, and efforts were made to build the capacity of frontline implementers and families in egg feeding and baby-friendly poultry. The unique approach through which the children were enabled to own chickens is also the strength of our trial that, we believe, helps sustain poultry production and egg-feeding behavior. The study duration might have limited our trial to see the intervention effect on LAZ. The wide age range of target children and the sample size was not ideal to assess the intervention effect on motor development, particularly hands and feet crawling, walking with assistance, standing alone and walking alone milestones. A further limitation was that gross motor milestones were collected by HDTLs, not experts in the field. We provided them with two days of practical training before they were first deployed for data collection and a skill-reinforcing training monthly. However, taking the nature of milestone assessment into account, the successive training we provided them with might not be good enough to gather quality data. Finally, we lacked information on children’s exposure to chicken feces and thus we could not ascertain the role of cage utilization in improving child health and nutrition.

## 5. Conclusions

This nutrition-sensitive poultry intervention that enabled children to be owners of chickens significantly improved their nutritional status and gross motor milestone development. The intervention was significantly effective, particularly in increasing weight-for-age and weight-for-height z-scores and the early attainment of running, kicking a ball and throwing ball motor skills. The odds of being underweight and stunting were significantly reduced. The increasing effect on the length-for-age z-score was also remarkable. Combined with its potential for sustainability, the results found in improving child growth and development make this model of nutrition-sensitive poultry intervention a plausible alternative to alleviate malnutrition, particularly in settings where animal source food intake is low. Implementation at a large scale for a longer period in the first two years of life is recommended. 

## Figures and Tables

**Table 1:** Baseline socio-economic and demographic characteristics and IYCF practices.

Description		Intervention (N = 127)	Control (N = 126)
		%	%
**Household characteristics**			
Main source of income	Farming	89.8	84.1
Main source of drinking water	Community tap water	100	97.6
**Livestock production**			
Poultry production	Chicken	20.5	26.2
Chicken care	Day cage/separated place	11.5	6.1
	Night shelter/cage	19.2	15.2
**Maternal Characteristics**			
Age in years	Mean age (SD)	27.3 (4.68)	27.5 (4.18)
Educational Status	Illiterate	66.1	54
	Read and write	26.8	34.1
	Formal education	7.1	11.9
Education on feeding eggs	Received	40.2	41.3
Awareness on disease risk of chicken feces	Aware	23.6	31.7
**Child Characteristics**			
Sex	Female *	36.2	54.8
Age (month)	Mean (SD)	10.9 (3.18)	11.4 (4.28)
**IYCF**			
Breastfeeding	Currently fed on breastmilk	98.4	96.8
Complementary food	Currently on complementary food	94.5	92.9
	Mean age of introduction: months (SD)	6.13 (0.59)	6.2 (0.69)
Egg intake history	Ever fed	51.2	45.2
% children fed with egg	24 h before survey	7.9	9.5
	The week before survey	23.6	27.8
Eggs consumed (per week per child)	Mean (SD)	0.23 (0.42)	0.29 (0.51)
Minimum dietary diversity (MDD)	Children fulfilling MDD	4.7	6.3
	Mean Dietary Diversity score (SD)	2.34 (0.97)	2.45 (0.94)

* Statistically significant (*p* < 0.05).

**Table 2:** Nutritional status of intervention and control groups at baseline and end line.

	Baseline		End Line		Significance Testing	
	Intervention (N = 122)	Control (N = 121)	Intervention (N = 122)	Control (N = 121)		
	Mean (SD)	Mean (SD)	Mean (SD)	Mean (SD)	*β* (95% CI) ^a^	*p*
WAZ	−1.12 (1.00)	−1.02 (1.17)	−0.20 (0.86)	−1.09 (1.05)	0.38 (0.13, 0.63)	0.003
LAZ	−1.32 (1.19)	−1.10 (1.48)	−1.04 (1.04)	−1.58 (1.25)	0.15 (−0.15, 0.44)	0.325
WHZ	−0.65 (0.97)	−0.63 (1.03)	0.43 (0.79)	−0.40 (0.89)	0.43 (0.21, 0.64)	<0.001
	N (%)	N (%)	N (%)	N (%)	OR (95% CI) ^b^	*p*
Underweight	22 (18)	24 (19.8)	6 (4.9)	22 (18.2)	0.46 (0.26, 0.84)	0.011
Stunting	35 (28.7)	37 (30.6)	41 (33.6)	50 (41.3)	0.58 (0.37, 0.91)	0.017
Wasting	11 (9)	10 (8.3)	1 (0.8)	6 (5)	0.52 (0.26, 1.05)	0.067

OR: Odds ratio; WAZ: Weight for age Z-score; LAZ: Length for age Z-score; WHZ: Weight for height Z-score ^a^ Calculated by GEE linear model with autoregressive correlation matrix, ^b^ Calculated by GEE binary logit adjusted for baseline corresponding Z-score.

**Table 3:** Comparison of mean age of attainment of gross motor development milestones among normal weight children (WAZ ≥ −2.0) at baseline in intervention and control groups.

Gross Motor Milestone	Number of Children	Age of Attainment Mean (SD) Months	*p* ^1^	CHR (95% CI)	*p*	AHR (95% CI) ^2^	*p*	AHR (95% CI) ^3^	*p*
Hands and knees crawling	IG = 6	10.93 (0.06)	0.753 *	1.22 (0.30–4.97)	0.784	1.03 (0.24–4.41) ^a^	0.970	0.94 (0.22–3.92)	0.928
	CG = 12	10.85 (0.41)							
Walking with assistance	IG = 28	12.24 (0.71)	0.721 **	0.90 (0.49–1.67)	0.740	0.90 (0.48–1.70) ^a^	0.751	0.85 (0.45–1.59)	0.606
	CG = 34	11.98 (0.67)							
Standing alone	IG = 58	13.54 (1.07)	0.943 *	0.99 (0.65–1.49)	0.945	0.98 (0.65–1.49) ^a^	0.922	1.02 (0.67–1.55)	0.944
	CG = 62	13.39 (1.12)							
Walking alone	IG = 89	15.15 (1.81)	0.317 *	1.09 (0.77–1.53)	0.623	1.18 (0.83–1.67)	0.369	1.19 (0.83–1.69)	0.342
	CG = 89	15.0 (1.97)							
Running	IG = 117	17.53 (1.70)	0.021 *	1.34 (0.99–1.82)	0.061	1.43 (1.05–1.95) ^a^	0.022	1.41 (1.04–1.93)	0.028
	CG = 110	17.96 (2.26)							
Kicking ball forward	IG = 124	18.96 (1.73)	0.027 *	1.32 (0.99–1.77)	0.061	1.37 (1.02–1.84)	0.036	1.39 (1.04–1.87)	0.027
	CG = 122	19.41 (2.38)							
Throwing ball overhead	IG = 113	20.90 (1.31)	0.046 **	1.29 (0.95–1.75)	0.099	1.34 (0.98– 1.82)	0.064	1.37 (1.01–1.86)	0.045
	CG = 96	21.18 (1.64)							
Jumping up	IG = 67	23.42 (0.70)	0.061 **	1.33 (0.91–1.94)	0.135	1.37 (0.94– 2.00)	0.106	1.38 (0.94–2.02)	0.099
	CG = 63	23.50 (0.83)							

IG = Intervention group; CG = Control group; CHR = Crude hazard ratio; AHR = Adjusted hazard ratio. Note: Crude and adjusted hazard ratios were computed by Cox regression. ^1^ Computed by Log-rank test. * Adjusted for baseline stunting. ** Adjusted for baseline co-morbidity of anemia and stunting. ^2^ Adjusted for baseline co-morbidity of anemia and stunting except those denoted by (^a^) that were adjusted for baseline stunting and anemia. ^3^ Computed by Cox-regression and adjusted for baseline co-morbidity of anemia and stunting, wasting and anemia.

## Data Availability

Data will be made available upon request.

## Supplementary Materials

The following supporting information can be downloaded at: https://www.mdpi.com/article/10.3390/ijerph192215305/s1, Supplemental Figure S1. Distribution of anthropometric z-scores among children in intervention and control groups at baseline and end-line (solid) compared with WHO standards (dashed); Supplemental Table S1. Comparison of intervention effect on nutritional status among treatment and control groups by GEE different correlation matrices.

## References

[1] United Nations Children’s Fund (UNICEF), World Health Organization, International Bank for Reconstruction and Development/World Bank (2021). Levels and Trends in Child Malnutrition: Key Findings of the 2021 Edition of the Joint Child Malnutrition Estimates.

[2] Ethiopian Public Health Institute (EPHI), ICF (2021). Ethiopia Mini Demographic and Health Survey 2019: Key Indicators.

[3] United Nations Children’s Fund (UNICEF), World Health Organization, International Bank for Reconstruction and Development/The World Bank (2020). Levels and Trends in Child Malnutrition: Key Findings of the 2020 Edition of the Joint Child Malnutrition Estimates.

[4] Amaha N.D. (2020). Ethiopian Progress towards Achieving the Global Nutrition Targets of 2025: Analysis of Sub-National Trends and Progress Inequalities. BMC Res. Notes.

[5] Global Nutrition Report Ethiopia Nutrition Profile. https://globalnutritionreport.org/resources/nutrition-profiles/africa/eastern-africa/ethiopia/.

[6] Ethiopian Public Health Institute (2013). Ethiopian National Food Consumption Survey.

[7] ICF Central Statistical Agency (CSA), ICF (2016). Ethiopia Demographic and Health Survey 2016.

[8] Réhault-Godbert S., Guyot N., Nys Y. (2019). The Golden Egg: Nutritional Value, Bioactivities, and Emerging Benefits for Human Health. Nutrients.

[9] Bragg M., Caswell B., Maleta K., Stewart C. (2020). Choline Intake in Malawian Children Aged 6–9 and 12–15 Months in an Egg Intervention Trial. Curr. Dev. Nutr..

[10] Caswell B., Arnold C., Lutter C., Maleta K., Stewart C. (2020). An Egg Feeding Intervention Increased Protein Quantity and Quality Among Young Malawian Children. Curr. Dev. Nutr..

[11] Iannotti L.L., Lutter C.K., Waters W.F., Gallegos Riofrío C.A., Malo C., Reinhart G., Palacios A., Karp C., Chapnick M., Cox K. (2017). Eggs Early in Complementary Feeding Increase Choline Pathway Biomarkers and DHA: A Randomized Controlled Trial in Ecuador. Am. J. Clin. Nutr..

[12] Papanikolaou Y., Fulgoni V.L. (2021). Increasing Egg Consumption at Breakfast Is Associated with Increased Usual Nutrient Intakes: A Modeling Analysis Using NHANES and the USDA Child and Adult Care Food Program School Breakfast Guidelines. Nutrients.

[13] Papanikolaou Y., Fulgoni V.L. (2019). Egg Consumption in U.S. Children Is Associated with Greater Daily Nutrient Intakes, Including Protein, Lutein + Zeaxanthin, Choline, α-Linolenic Acid, and Docosahexanoic Acid. Nutrients.

[14] Iannotti L.L., Lutter C.K., Stewart C.P., Gallegos Riofrío C.A., Malo C., Reinhart G., Palacios A., Karp C., Chapnick M., Cox K. (2017). Eggs in Early Complementary Feeding and Child Growth: A Randomized Controlled Trial. Pediatrics.

[15] Stewart C.P., Caswell B., Iannotti L., Lutter C., Arnold C.D., Chipatala R., Prado E.L., Maleta K. (2019). The Effect of Eggs on Early Child Growth in Rural Malawi: The Mazira Project Randomized Controlled Trial. Am. J. Clin. Nutr..

[16] Omer A., Mulualem D., Classen H., Vatanparast H., Whiting S.J. (2019). Promotion of Egg and Eggshell Powder Consumption on the Nutritional Status of Young Children in Ethiopia. Int. J. Food Sci. Nutr. Res..

[17] McKune S.L., Stark H., Sapp A.C., Yang Y., Slanzi C.M., Moore E.V., Omer A., Wereme N’Diaye A. (2020). Behavior Change, Egg Consumption, and Child Nutrition: A Cluster Randomized Controlled Trial. Pediatrics.

[18] Prado E.L., Maleta K., Caswell B.L., George M., Oakes L.M., DeBolt M.C., Bragg M.G., Arnold C.D., Iannotti L.L., Lutter C.K. (2020). Early Child Development Outcomes of a Randomized Trial Providing 1 Egg Per Day to Children Age 6 to 15 Months in Malawi. J. Nutr..

[19] Alderman H., Gilligan D.O., Leight J., Mulford M., Tambet H. (2022). The Role of Poultry Transfers in Diet Diversity: A Cluster Randomized Intent to Treat Analysis. Food Policy.

[20] Broaddus-Shea E.T., Manohar S., Thorne-Lyman A.L., Bhandari S., Nonyane B.A.S., Winch P.J., West K.P. (2020). Small-Scale Livestock Production in Nepal Is Directly Associated with Children’s Increased Intakes of Eggs and Dairy, But Not Meat. Nutrients.

[21] Mosites E., Aol G., Otiang E., Bigogo G., Munyua P., Montgomery J.M., Neuhouser M.L., Palmer G.H., Thumbi S.M. (2017). Child Height Gain Is Associated with Consumption of Animal-Source Foods in Livestock-Owning Households in Western Kenya. Public Health Nutr..

[22] Bardosh K.L., Hussein J.W., Sadik E.A., Hassen J.Y., Ketema M., Ibrahim A.M., McKune S.L., Havelaar A.H. (2020). Chicken Eggs, Childhood Stunting and Environmental Hygiene: An Ethnographic Study from the Campylobacter Genomics and Environmental Enteric Dysfunction (CAGED) Project in Ethiopia. One Health Outlook.

[23] Headey D., Nguyen P., Kim S., Rawat R., Ruel M., Menon P. (2017). Is Exposure to Animal Feces Harmful to Child Nutrition and Health Outcomes? A Multicountry Observational Analysis. Am. J. Trop. Med. Hyg..

[24] Headey D., Hirvonen K. (2016). Is Exposure to Poultry Harmful to Child Nutrition? An Observational Analysis for Rural Ethiopia. PLoS ONE.

[25] Syed S., Ali A., Duggan C. (2016). Environmental Enteric Dysfunction in Children: A Review. J. Pediatr. Gastroenterol. Nutr..

[26] Passarelli S., Ambikapathi R., Gunaratna N.S., Madzorera I., Canavan C.R., Noor R.A., Tewahido D., Berhane Y., Sibanda S., Sibanda L.M. (2021). The Role of Chicken Management Practices in Children’s Exposure to Environmental Contamination: A Mixed-Methods Analysis. BMC Public Health.

[27] Omer A., Mulualem D., Classen H., Vatanparast H., Whiting S.J. (2018). A Community Poultry Intervention to Promote Egg and Eggshell Powder Consumption by Young Children in Halaba Special Woreda, SNNPR, Ethiopia. J. Agric. Sci..

[28] Omer A., Hailu D., Whiting S.J. (2022). Egg Consumption of Children under Two Years of Age through a Child-Owned Poultry and Nutrition Intervention in Rural Ethiopia: A Community-Based Randomized Controlled Trial. J. Agric. Food Res..

[29] World Health Organization, United Nations Children’s Fund (UNICEF) (2019). Recommendations for Data Collection, Analysis and Reporting on Anthropometric Indicators in Children under 5 Years Old.

[30] WHO (2006). Multicentre Growth Reference Study Group WHO Motor Development Study: Windows of Achievement for Six Gross Motor Development Milestones. Acta Paediatr. Suppl..

[31] Wijnhoven T.M., de Onis M., Onyango A.W., Wang T., Bjoerneboe G.-E.A., Bhandari N., Lartey A., al Rashidi B. (2004). Assessment of Gross Motor Development in the WHO Multicentre Growth Reference Study. Food Nutr. Bull..

[32] Frankenburg W.K., Dodds J., Archer P., Shapiro H., Bresnick B. (1992). The Denver II: A Major Revision and Restandardization of the Denver Developmental Screening Test. Pediatrics.

[33] Cheesbrough M. (2005). District Laboratory Practice in Tropical Countries. Part 1.

[34] World Health Organization (2018). WHO Anthro Survey Analyser: Software for Analysing Survey Anthropometric Data for Children under 5 Years of Age. Built-in Software Edition.

[35] Pasqualino M., Shaikh S., Hossain M.I., Islam M.T., Ali H., Haque R., Ayesha K., Wu L., Schuh H., Dyer B. (2020). The Effect of Eggs on Growth Among Infants 6–12 Months of Age in Rural Bangladesh: A Cluster Randomized Controlled Trial. Curr. Dev. Nutr..

[36] Mekonnen T.C., Tadesse S.E., Dawed Y.A., Cherie N., Abebe H., Shumye G., Mohammed F., Hussien A. (2022). The Role of Nutrition-sensitive Agriculture Combined with Behavioral Interventions in Childhood Growth in Ethiopia: An Adequacy Evaluation Study. Health Sci. Rep..

[37] Dumas S.E., Lewis D., Travis A.J. (2018). Small-Scale Egg Production Centres Increase Children’s Egg Consumption in Rural Zambia. Matern. Child Nutr..

[38] Gupta A., Kalaivani M., Gupta S.K., Rai S.K., Nongkynrih B. (2016). The Study on Achievement of Motor Milestones and Associated Factors among Children in Rural North India. J. Family Med. Prim. Care.

[39] Kariger P.K., Stoltzfus R.J., Olney D., Sazawal S., Black R., Tielsch J.M., Frongillo E.A., Khalfan S.S., Pollitt E. (2005). Iron Deficiency and Physical Growth Predict Attainment of Walking but Not Crawling in Poorly Nourished Zanzibari Infants. J. Nutr..

[40] Siegel E.H., Stoltzfus R.J., Kariger P.K., Katz J., Khatry S.K., LeClerq S.C., Pollitt E., Tielsch J.M. (2005). Growth Indices, Anemia, and Diet Independently Predict Motor Milestone Acquisition of Infants in South Central Nepal. J. Nutr..

[41] Vaida N. (2013). Development of Children during First 2 Years of Life. Stud. Home Community Sci..

[42] Stewart C.P., Kariger P., Fernald L., Pickering A.J., Arnold C.D., Arnold B.F., Hubbard A.E., Dentz H.N., Lin A., Meerkerk T.J. (2018). Effects of Water Quality, Sanitation, Handwashing, and Nutritional Interventions on Child Development in Rural Kenya (WASH Benefits Kenya): A Cluster-Randomised Controlled Trial. Lancet Child Adolesc. Health.

[43] Tofail F., Fernald L.C., Das K.K., Rahman M., Ahmed T., Jannat K.K., Unicomb L., Arnold B.F., Ashraf S., Winch P.J. (2018). Effect of Water Quality, Sanitation, Hand Washing, and Nutritional Interventions on Child Development in Rural Bangladesh (WASH Benefits Bangladesh): A Cluster-Randomised Controlled Trial. Lancet Child Adolesc. Health.

[44] Frongillo E.A., Nguyen P.H., Saha K.K., Sanghvi T., Afsana K., Haque R., Baker J., Ruel M.T., Rawat R., Menon P. (2017). Large-Scale Behavior-Change Initiative for Infant and Young Child Feeding Advanced Language and Motor Development in a Cluster-Randomized Program Evaluation in Bangladesh. J. Nutr..

[45] Prado E.L., Adu-Afarwuah S., Lartey A., Ocansey M., Ashorn P., Vosti S.A., Dewey K.G. (2016). Effects of Pre- and Post-Natal Lipid-Based Nutrient Supplements on Infant Development in a Randomized Trial in Ghana. Early Hum. Dev..

[46] Prado E.L., Abbeddou S., Yakes Jimenez E., Somé J.W., Ouédraogo Z.P., Vosti S.A., Dewey K.G., Brown K.H., Hess S.Y., Ouédraogo J.-B. (2015). Lipid-Based Nutrient Supplements Plus Malaria and Diarrhea Treatment Increase Infant Development Scores in a Cluster-Randomized Trial in Burkina Faso. J. Nutr..

[47] Matias S.L., Mridha M.K., Tofail F., Arnold C.D., Khan M.S.A., Siddiqui Z., Ullah M.B., Dewey K.G. (2017). Home Fortification during the First 1000 d Improves Child Development in Bangladesh: A Cluster-Randomized Effectiveness Trial. Am. J. Clin. Nutr..

[48] Prado E.L., Maleta K., Ashorn P., Ashorn U., Vosti S.A., Sadalaki J., Dewey K.G. (2016). Effects of Maternal and Child Lipid-Based Nutrient Supplements on Infant Development: A Randomized Trial in Malawi. Am. J. Clin. Nutr..

[49] Zulkarnaen Z. (2019). The Influence of Nutritional Status on Gross and Fine Motor Skills Development in Early Childhood. Asian Soc. Sci..

[50] Workie S.B., Mekonen T., Mekonen T.C., Fekadu W. (2020). Child Development and Nutritional Status in 12–59 Months of Age in Resource Limited Setting of Ethiopia. J. Health Popul. Nutr..

[51] Iannotti L., Jean Louis Dulience S., Wolff P., Cox K., Lesorogol C., Kohl P. (2016). Nutrition Factors Predict Earlier Acquisition of Motor and Language Milestones among Young Children in Haiti. Acta Paediatr..

[52] Faber M., Malan L., Kruger H.S., Asare H., Visser M., Mukwevho T., Ricci C., Smuts C.M. (2022). Potential of Egg as Complementary Food to Improve Nutrient Intake and Dietary Diversity. Nutrients.

[53] Caswell B.L., Arnold C.D., Lutter C.K., Iannotti L.L., Chipatala R., Werner E.R., Maleta K.M., Stewart C.P. (2021). Impacts of an Egg Intervention on Nutrient Adequacy among Young Malawian Children. Matern. Child Nutr..

[54] Bragg M.G., Prado E.L., Stewart C.P. (2022). Choline and Docosahexaenoic Acid during the First 1000 Days and Children’s Health and Development in Low- and Middle-Income Countries. Nutr. Rev..

[55] Cohen Kadosh K., Muhardi L., Parikh P., Basso M., Jan Mohamed H.J., Prawitasari T., Samuel F., Ma G., Geurts J.M.W. (2021). Nutritional Support of Neurodevelopment and Cognitive Function in Infants and Young Children—An Update and Novel Insights. Nutrients.

[56] Schwarzenberg S.J., Georgieff M.K., Daniels S., Corkins M., Golden N.H., Kim J.H., Lindsey C.W., Magge S.N. (2018). Advocacy for Improving Nutrition in the First 1000 Days to Support Childhood Development and Adult Health. Pediatrics.

[57] Georgieff M.K. (2011). Long-Term Brain and Behavioral Consequences of Early Iron Deficiency. Nutr. Rev..

[58] Haas J.D., Rivera-Dommarco J. (2020). The Effects of Improved Nutrition in Early Childhood on Adolescent and Early Adulthood Body Size, Composition, Maturity, and Function: Results from the First INCAP Follow-Up Study. Food Nutr. Bull..

[59] Wozniak J.R., Fink B.A., Fuglestad A.J., Eckerle J.K., Boys C.J., Sandness K.E., Radke J.P., Miller N.C., Lindgren C., Brearley A.M. (2020). Four-Year Follow-up of a Randomized Controlled Trial of Choline for Neurodevelopment in Fetal Alcohol Spectrum Disorder. J. Neurodev. Disord..

[60] Oberhelman R.A., Gilman R.H., Sheen P., Cordova J., Zimic M., Cabrera L., Meza R., Perez J. (2006). An Intervention-Control Study of Corralling of Free-Ranging Chickens to Control Campylobacter Infections Among Children in a Peruvian Periurban Shantytown. Am. J. Trop. Med. Hyg..

[61] Omer A. (2020). Poultry Interventions and Child Nutrition Status in Low-Income Countries. Afr. J. Food Agric. Nutr. Dev..

[62] Iannotti L.L., Chapnick M., Nicholas J., Gallegos-Riofrio C.A., Moreno P., Douglas K., Habif D., Cui Y., Stewart C., Lutter C.K. (2020). Egg Intervention Effect on Linear Growth No Longer Present after Two Years. Matern. Child Nutr..

