# Supplementary material for: Effect of a Child-Owned Poultry Intervention Providing Eggs on Nutrition Status and Motor Skills of Young Children in Southern Ethiopia: A Cluster Randomized and Controlled Community Trial
Source: Int J Environ Res Public Health. 2022 Nov 19;19(22):15305. doi: 10.3390/ijerph192215305 (PMC9690635; doi:10.3390/ijerph192215305)
Supplement: Supplementary file 1 [file ijerph-19-15305-s001.zip › ijerph-2024707-supplementary.pdf]

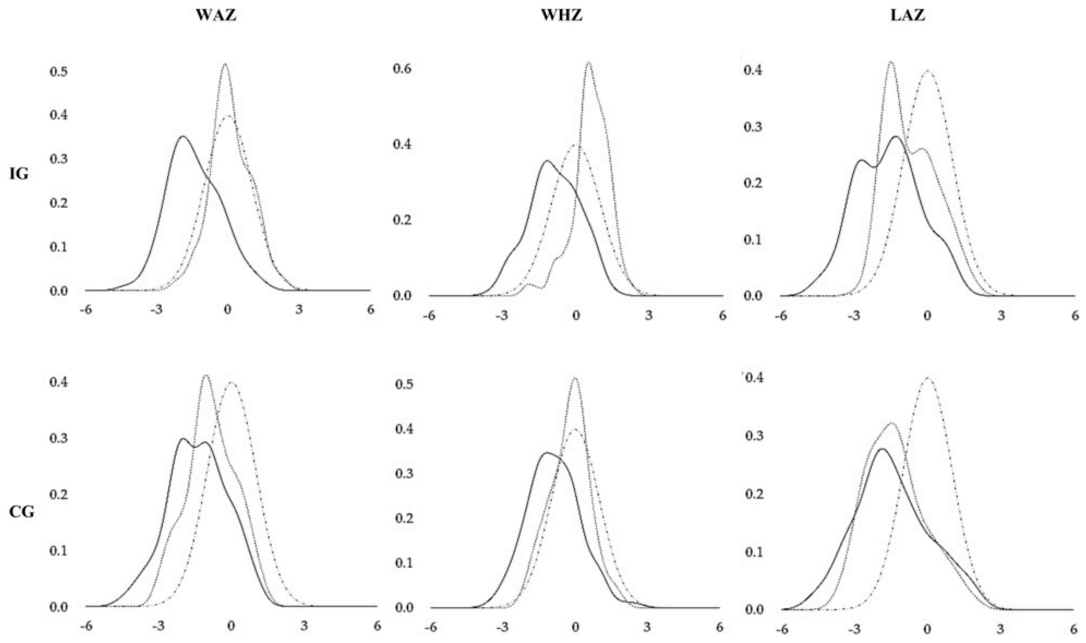

Supplemental Figure S1. Distribution of anthropometric z-scores among children compared with WHO standards (dashed). IG = Intervention group; CG = Control group. WAZ= weight for age Z-score; WHZ = weight for height (length) Z-score; LAZ = length for age Z-score. Baseline is solid line, end line is dotted line.

Supplemental Table S1. Comparison of intervention effect on nutritional status among treatment and control groups by GEE different correlation matrices.

	Baseline		End line		Significance testing					
	**Interven-tion (N=122)**	**Control (N=121)**	**Interven-tion (N=122)**	**Control (N=121)**	**Correlation matrix: AR**		**Correlation matrix: In-dependent**		**Correlation matrix: Ex-changeable**	
	**Mean (SD)**	**Mean (SD)**	**Mean (SD)**	**Mean (SD)**	***β* (95% CI)[a]**	***p***	***β* (95% CI)[a]**	***p***	***β* (95% CI)[a]**	***p***
WAZ	-1.12 (1.00)	-1.02 (1.17)	-0.20 (0.86)	-1.09 (1.05)	0.38 (0.13, 0.63)	0.003	0.27 (0.02, 0.52)	0.033	0.27 (0.02, 0.52)	0.034
LAZ	-1.32 (1.19)	-1.10 (1.48)	-1.04 (1.04)	-1.58 (1.25)	0.15 (-0.15, 0.44)	0.325	0.10 (-0.18, 0.39)	0.473	0.11 (-0.17, 0.39)	0.450
WHZ	-0.65 (0.97)	-0.63 (1.03)	0.43 (0.79)	-0.40 (0.89)	0.43 (0.21, 0.64)	<0.001	0.45 (0.24, 0.66)	<0.001	0.45 (0.24, 0.67)	<0.001
	**N (%)**	**N (%)**	**N (%)**	**N (%)**	**OR (95% CI)[b]**	***p***	**OR (95% CI)[b]**	***p***	**OR (95% CI)[b]**	***p***
Under-weight	22 (18)	24 (19.8)	6 (4.9)	22 (18.2)	0.46 (0.26, 0.84)	0.011	0.52 (0.29, 0.94)	0.031	0.52 (0.28, 0.94)	0.03
Stunting	35 (28.7)	37 (30.6)	41 (33.6)	50 (41.3)	0.58 (0.37, 0.91)	0.017	0.58 (0.37, 0.91)	0.019	0.58 (0.37, 0.91)	0.018
Wasting	11 (9)	10 (8.3)	1 (0.8)	6 (5)	0.52 (0.26, 1.05)	0.067	0.52 (0.26, 1.04)	0.066	0.52 (0.26, 1.05)	0.068

AR: Auto-regressive; OR: Odds ratio; WAZ: Weight for age Z-score; LAZ: Length for age Z-score; WHZ: Weight for height Z-score. [a] Calculated by GEE linear model. [b] Calculated by GEE binary logit adjusted for baseline corresponding Z-score.
